# Simulations of Prebiotic Chemistry under Post-Impact Conditions on Titan

**DOI:** 10.3390/life3040538

**Published:** 2013-12-17

**Authors:** Carol Turse, Johannes Leitner, Maria Firneis, Dirk Schulze-Makuch

**Affiliations:** 1 School of the Environment, Washington State University, Pullman, WA 99164, USA, E-Mail: dirksm@wsu.edu; 2 Research Platform on ExoLife, University of Vienna, Türkenschanzstraße 17, Vienna 1180, Austria; E-Mails: johannes.leitner@univie.ac.at (J.L.); maria.firneis@univie.ac.at (M.F.)

**Keywords:** prebiotic chemistry, Miller-Urey, Titan, amino acids

## Abstract

The problem of how life began can be considered as a matter of basic chemistry. How did the molecules of life arise from non-biological chemistry? Stanley Miller’s famous experiment in 1953, in which he produced amino acids under simulated early Earth conditions, was a huge leap forward in our understanding of this problem. Our research first simulated early Earth conditions based on Miller’s experiment and we then repeated the experiment using Titan post-impact conditions. We simulated conditions that could have existed on Titan after an asteroid strike. Specifically, we simulated conditions after a potential strike in the subpolar regions of Titan that exhibit vast methane-ethane lakes. If the asteroid or comet was of sufficient size, it would also puncture the icy crust and bring up some of the subsurface liquid ammonia-water mixture. Since, O’Brian, Lorenz and Lunine showed that a liquid water-ammonia body could exist between about 10^2^–10^4^ years on Titan after an asteroid impact we modified our experimental conditions to include an ammonia-water mixture in the reaction medium. Here we report on the resulting amino acids found using the Titan post-impact conditions in a classical Miller experimental reaction set-up and how they differ from the simulated early Earth conditions.

## 1. Introduction

In his famous experiment, Stanley Miller added supposed components of the early Earth’s atmosphere to a closed system containing a sample “early ocean” as well as electrodes to simulate lightening [1]. His experimental reducing atmosphere contained methane, hydrogen, ammonia and water vapor. After seven days, Miller detected amino acids in the ocean flask in this closed system. Specifically, the amino acids found were alanine, glycine and aspartic acid [1]. Although several different groups had attempted simple organic synthesis under primitive conditions beginning in the early 20th century [2,3,4], Miller was the first to put the synthesis in the perspective of Darwin’s “prebiotic soup”. Essentially, he initiated the whole field of the origin of life as a topic of serious scientific investigation. Even though further research has shown that the early atmosphere on the Earth may not have been as rich in methane (and thus reducing) as Miller thought [5,6], with carbon dioxide playing a larger role, localized reducing environments could have existed near volcanic activity [7]. Replicates of the Miller-Urey experiment with carbon dioxide instead of methane have shown that amino acids are much harder to make in the carbon dioxide rich atmosphere [8] However, Miller’s experiments remain relevant as a starting point and inspiration in the investigations of the origin of life. Variations of Miller’s experiments, some completed by Miller himself, have been completed that include aspects of hydrothermal vents, neutral atmospheres, reducing H_2_S atmospheres, as well as volcanic conditions [9]. In each of these variations amino acids or organic precursors of amino acids are produced at some level.

Our research first simulated early Earth conditions based on Miller’s experiment and then reproduced it using conditions that may occur on Saturn’s largest moon Titan. Specifically we modeled conditions that could occur after a large impact on Titan. We simulated conditions that could have existed on Titan after an asteroid strike [10]. Specifically, we simulated conditions after a potential strike in the subpolar regions of Titan that exhibit vast methane-ethane lakes. If the asteroid or comet was of sufficient size, it would also puncture the icy crust and bring up some of the subsurface liquid ammonia-water mixture. Since O’Brien, Lorenz and Lunine [11] showed that a liquid water-ammonia body could exist for up to thousands of years on Titan after an asteroid impact we modified our experimental conditions to include the ammonia-water liquid in the reaction medium. Static discharge or lightening are expected on Titan due to atmospheric processes such as convective storms [12,13] and as side effect of the asteroid impact. Other discharge experiments simulating the chemical evolution on Titan have shown that it is possible to make carbon, hydrogen and nitrogen compounds that are important precursors to amino acids [14]. Here, we report on the resulting amino acids found using Titan post-impact conditions in a classical Miller experimental reaction set-up and how they differ from the simulated early Earth conditions.

## 2. Experimental Section

### 2.1. Experimental Apparatus and Cleaning

**Figure 1:**
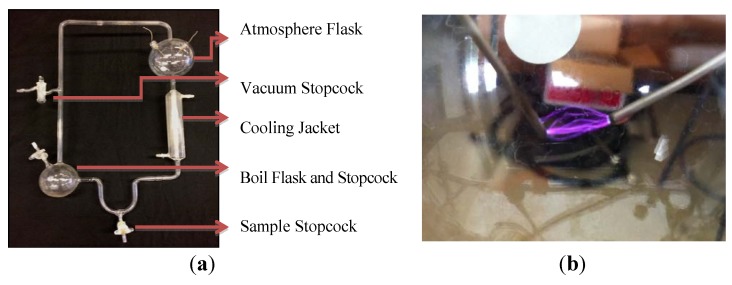
(**a**) Custom built reaction apparatus used to simulate early Earth and Titan post-impact conditions; (**b**) Spark gap inside the atmospheric flask.

An apparatus was built replicating the original apparatus used by Stanley Miller in 1953 [1]. This apparatus contains a 250 mL round bottom flask attached to a 1000 mL atmospheric flask that is in turn attached to a u-bend sample port via a water-jacketed condensation tube (Figure 1a). A heating jacket attached to a Staco variable transformer surrounds the 250 mL round bottom flask and there are several sample ports and stopcocks along the perimeter of the apparatus. Most samples are taken using the stopcock attached to the bottom u-bend. An electrical spark is applied to the leads on the atmospheric flask using a high voltage (20,000–45,000 volts) Tesla coil attached to an analog timer to provide a random on/off cycles. This Tesla coil then initiates a spark in the atmospheric flask (Figure 1b). Before each experimental run (and between each subsequent run) the apparatus was cleaned with approximately 150 mL of a hot 10% sodium hydroxide solution followed by 5 rinses each with about 70 mL of Nanopure water. 150 mL of a dilute sulfuric acid was then added followed by 5 rinses each with Nanopure water. The apparatus was then filled with 190 mL of ddH_2_O and boiled for 24 h to thoroughly flush the system. The water was then removed and another rinse with 200 mL of ddH_2_O was completed. 

### 2.2. Experimental Conditions: Early Earth

After the cleaning procedures were completed approximately 200 mL of ddH_2_O was added back to the round bottom flask. Vacuum was applied for 10 min to purge the solution of gases. Hydrogen gas was then added to a pressure of 1 bar followed by application of vacuum for another 10 min. The vacuum purge and hydrogen flush procedure was repeated 3 times. After the final vacuum purge, gases were added in the following order to the following pressures: (1) hydrogen gas, 0.2–0.3 bar, (2) methane gas, 0.2–0.3 bar and (3) ammonia gas 0.2–0.3 bar. The total pressure inside the apparatus approached 1 bar. All stopcocks were then closed and a cooling water bath was applied to the condensation tube. Heat was applied using a Staco variable transformer and the solution was brought to a constant boil. After a constant boil was achieved the Tesla coil attached to a manual timer was used to apply a spark (up to 45,000 volts) to the electrode in the upper chamber (Figure 1b). The spark was applied in a random manner using an analog timer attached to the electrical outlet. The experiment was run for 7 days. Samples were taken at the end of 7 days using the sample stopcock (Figure 1a). These experimental conditions were repeated three times for a total of three early Earth samples. Samples were stored in screw top GC/MS sample vials at +4 °C until analysis was completed.

### 2.3. Experimental Conditions: Titan Post-Impact, Time Course Experiment

The round bottom flask was filled with 200 mL of 30% aqueous ammonia and the apparatus was placed under vacuum for 10 min to purge the solution of gases. The system was then flushed with hydrogen gas and placed under vacuum three times (10 min each time) to completely purge the system and solution. Gases were then added in the following order to the following pressures: hydrogen gas to 0.05 bar, methane gas to 1.0 bar and ammonia to 0.45 bar (~1.5 bar total). The water was then brought to a boil and the spark was applied using the Tesla coil up to a maximum of 45,000 volts (Figure 1b). The spark was applied in a random manner using an analog timer attached to the electrical outlet. The apparatus was run for 7 days and samples were taken over a time course (Table 1). Samples were taken at the beginning of the experiment (sample 1), 2 days post-application of spark (sample 2), 4 days post-application of spark (sample 3) and 7 days post-application of spark (sample 4). The samples taken at the beginning of the experiment (sample 1), 4 days post-spark application (sample 3) and 7 days post-spark application (sample 4) were taken using the sample stopcock (Figure 1a). The samples taken at 2 days (sample 2) were taken using the vacuum stopcock because a light pink color was noted in the reaction mixture in that area at 2 days post-spark application. This pink color was apparent at the sample stopcock from day four onward. These conditions were repeated three times resulting in three sets of time-course Titan post-impact samples. Samples were stored in screw top GC/MS sample vials at +4 °C until analysis was completed.

**Table 1:** Sample number, identification and location of sample in the apparatus.

Sample	Identification	Sample Location
Sample 1	Starting mixture of 30% aqueous ammonia	Sample Stopcock
Sample 2	2 days post-spark application	Vacuum Stopcock
Sample 3	4 days post-spark application	Sample Stopcock
Sample 4	7 days post-spark application	Sample Stopcock

### 2.4. Sample Analysis

All samples were run on a Waters MALDI Q-TOF Premier Micromass instrument in ESI+ Q-TOF W mode using direct injection. The injection method was direct using a 1 mL GC syringe coupled to a small motor so that the sample was injected continuously over a period of 10 min. All samples were diluted 1:10 in water before injection. A solution of 10% formic acid/0.1M sodium hydroxide/acetonitrile at a ratio of 1/1/8 was used to calibrate the instrument. The peaks from each sample with an intensity value above 15% were again run in MS-MS mode to help with identification and analysis. Post-run processing of samples was done on the MassLynx software from Waters Laboratories Informatics and included peak smoothing using the Savitzky Golay method and peak centering. Both processes reduced the background and limited peak widths to a value of 6 at half height.

The peak lists were then annotated based on the relative intensity of the peak. For example, in Titan time course sample 1 only peaks with relative intensities above 3 were considered. In other samples the background was such that only peaks with relative intensities above the background noise were considered. For example, in Titan time course sample 2, the background was such that only peaks with relative intensities over 40 were considered in the analysis.

After each file was annotated, the metabolite MS and MS-MS database from the Scripps Center for Metabolomics was used to search for peak matches [15]. The cut-off point for peak matches was 10 ppm. Peaks in the MS spectra were also identified by hand using the distances between peaks and known starting solutions as a guide.

## 3. Results and Discussion

### 3.1. Early Earth Conditions

The amino acids observed from the early Earth conditions are very similar to the amino acids seen in the original Miller experiment and in several follow-up experiments [1,9]. In Stanley Miller’s original experiment he found alanine (α and β), glycine and aspartic acid. As it is given in Table 2, we also found these three amino acids as well as several others. However, the main components of the reaction mixture, based on peak abundance, were alanine, glycine and aspartic acid.

**Table 2:** Amino acids found under early Earth experimental conditions.

Residue Mass (*m/z*) (monoisotopic, MH^+1^)	Peak Identification	Chemical Formula
72.03712	Alanine	C_3_H_5_NO
58.02147	Glycine	C_2_H_3_NO
116.02695	Aspartic Acid	C_4_H_5_NO_3_
114.08407	Isoleucine	C_6_H_11_NO
88.03203	Serine	C_3_H_5_NO_2_
102.04768	Threonine	C_4_H_7_NO_2_
113.08407	Leucine	C_6_H_11_NO
115.04293	Asparagine	C_4_H_6_N_2_O_2_
129.09497	Lysine	C_6_H_12_N_2_O
100.06842	Valine	C_5_H_9_NO
130.0426	Glutamic Acid	C_5_H_7_NO_3_
148.06842	Phenylalanine	C_9_H_9_NO
98.05277	Proline	C_5_H_7_NO

### 3.2. Titan Post-Impact Conditions, Time Course Experiment

**Figure 2:**
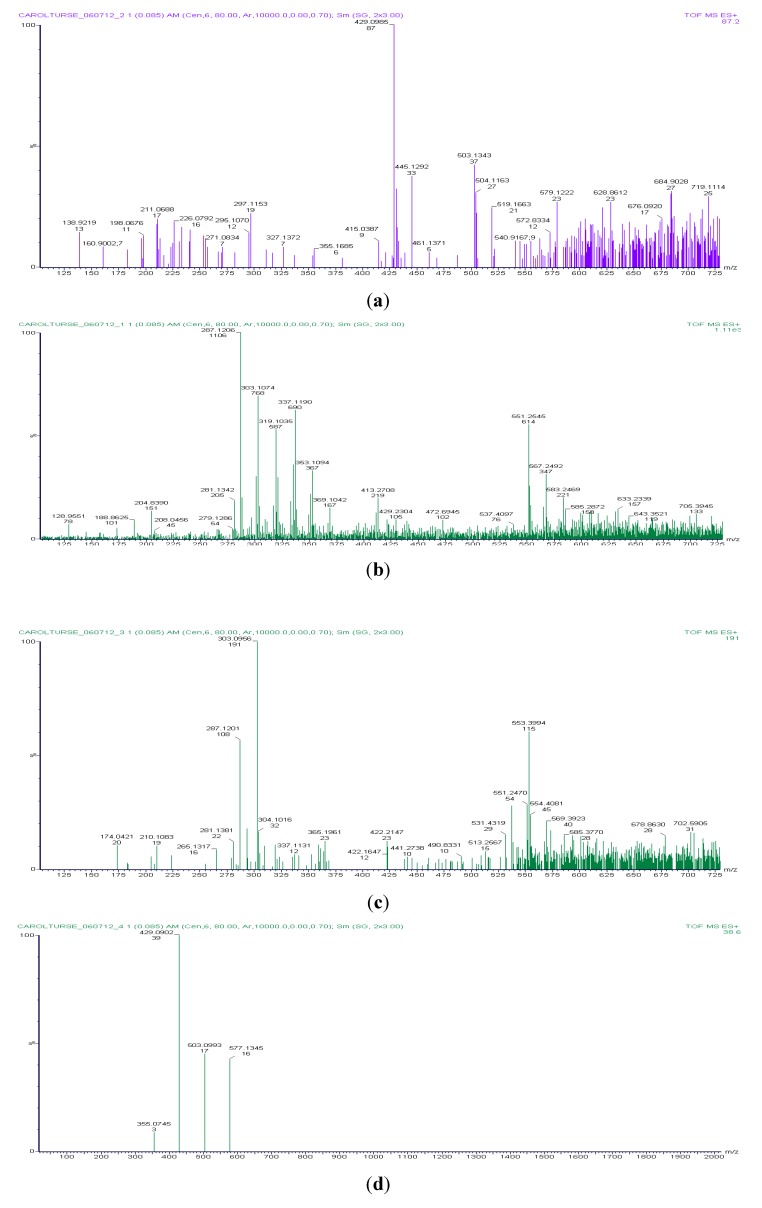
Representative MS Spectra from the Titan Time Course Experiments. All time points are the result of three independent replicates. (**a**) Sample 1, starting solution of 30% aqueous ammonia; (**b**) Sample 2, joint, 2 days post spark application; (**c**) Sample 3, U-bend, 4 days post spark application; (**d**) Sample 4, boil flask, 7 days post spark application.

The Titan time course experiments produced a range of amino acids as seen in Table 3 below. All of the reaction mixtures, except for the starting solution, were light pink in color, as opposed to the red-brown mixtures seen for the early Earth conditions. Miller attributed the red-brown color in the reaction mixture to organic compounds adsorbing to the colloidal silica from the reaction vessel [1]. It is important to note that he also observed a light pink color prior to the final red-brown of the reaction mixture. Representative spectra of all four time points are shown in Figure 2. Recall that a total of four samples were taken over the course of the 7 day Titan time course experiment. Sample 1 consisted of the starting solution of 30% aqueous ammonia, sample 2 was taken from the joint prior to the atmospheric chamber at 2 days post spark application, sample 3 was taken from the U-bend joint 4 days post spark application and sample 4 was taken from the boil flask 7 days post spark application.

**Table 3:** Amino Acids found in the Titan Time Course Experiments. All time points listed represent three independent replicates. Shaded green boxes in samples 2, 3 and 4 represent amino acids found in common between all three time points. The results from the early Earth condition experiments are included for reference.

	Time Course Sample Number	1	2	3	4	Early Earth Conditions
Compound Identification, relative abundance (%)		Ammonia (30% aqueous)	Glutamine, <10%	Histidine, 50%	Larger ring structures only, no single amino acids	Glycine
			Alanine, 25%	Aspartic Acid, <10%		Alanine
			Tyrosine, <10%	Tryptophan, <10%		Aspartic Acid
			Serine, <10%	Serine, <10%		Serine
			Phenylalanine, 50%	Phenylalanine, 25%		Phenylalanine
			Glutamic acid, <10%	Glutamic acid, <10%		Glutamic acid
			Arganine, <10%	Arganine, <10%		Asparagine
			Leucine, <10%	Leucine, <10%		Leucine
			Isoleucine, <10%	Isoleucine, <10%		Isoleucine
			Valine, <10%	Valine, <10%		Valine
				Proline, <10%		Proline
						Lysine
						Threonine

#### 3.2.1. Titan Sample 1, Starting Solution of 30% Aqueous Ammonia

The starting solution of 30% aqueous ammonia is shown in the representative spectrum in Figure 2. The peak gaps between adjacent peaks correspond to the molecular weight of nitrogen, hydrogen and oxygen in the positive ion mode. For example, if you consider peaks 303.1074 and 287.1206 you see a difference of 15.9868, which corresponds to nitrogen plus hydrogen. Since the solution is aqueous, hydroxide ion peaks are also common.

#### 3.2.2. Titan Sample Solution 2

These samples were taken 2 days after the experiment was started when we noticed a pink-red clear liquid pooling near the vacuum stopcock joint area prior to the atmospheric chamber. The joint area is located at the stopcock that leads to the inlet port for the gases used in the experiment. The amino acids in this sample are found in complexes of two to three amino acids (mostly groups of two). Specifically, glutamine, alanine, tyrosine, serine and phenylalanine were found in groups of two to three while glutamic acid, arginine, leucine, isoleucine and valine were found in separate groups of two to three amino acids each. The higher *m/z* peaks on the spectra represented multiple ring-type structures and are mostly occluded by the higher background.

#### 3.2.3. Titan Sample Solution 3

These samples were taken 4 days after the experiment started and had a slight pink color but had not reached the bright pink color seen at the end of 7 days. Samples were collected from the sample stopcock at the bottom of the apparatus. The amino acids were found in complexes of two to three amino acids. In this sample, arginine, proline, serine, aspartic acid, leucine, valine, isoleucine, glutamic acid, tryptophan, phenylalanine and histidine were seen in groups of two to three amino acids each. The most amino acids were identified from sample 3 as well as the most number of two to three amino acids groupings. The higher *m/z* peaks contained multiple ring-type structures and were mostly occluded by the higher background.

#### 3.2.4. Titan Sample Solution 4: Sample Taken from the Boil Flask

These samples were taken at the end of the 7 day experiments from the 250 mL round bottom flask using the sample stopcock. The samples were all a light pink in color. In sample 4 no amino acids were identified but several large ring structures were present. These structures included proanthocyanidin, alpha-L-rhamnopyranosyl-(1->2)-beta-D-galactopyranosyl-(1->2)-betva-D-glucuronopyranoside and a diethylstilbestrol diphosphate-like compound. All of these compounds have two or more benzene-like ring structures. The identification of a diphosphate-like compound was of some concern since no phosphorus was added to the reaction mixture. However, this could be explained by some carry-over from the cleaning solutions used on the Miller-Urey apparatus or as a lack in the database of spectra. Since the diphosphate compound didn’t appear on the spectra for samples 2 and 3, and since it did not appear in the starting solution, the problem is most likely one with the database.

### 3.3. Discussion

We were successful in replicating the original Miller-Urey early Earth conditions and produced many of the same amino acids seen by Stanley Miller. In particular we identified alanine, glycine and aspartic acid just as Miller did. Our experiments also identified several more amino acids than Miller (Table 2), which can be attributed to advanced analysis techniques.

In contrast, in the Titan time course samples we see an intriguing pattern emerge. Amino acids start to be produced 2 days after the application of spark and continue to increase in structural complexity (groups of two amino acids and then groups of three amino acids) up to 4 days after the spark is applied. Samples taken 2 days after the start of the experiment were taken from the vacuum stopcock because this is where we noticed a distinct pink-red clear solution. This pink color could be due to organonitrogen, like the pink-orange haze on Titan that is due to organic photochemistry in the atmosphere [16]. This pink color could also be similar to the pink color observed by Miller in his apparatus after a few days of running the experiment [1]. He attributed the pink-red color to organic compounds adsorbing to the colloidal silica from the reaction vessel and noticed that the pink color eventually turned into a dark red-brown color. Based on the spectra from day 2 forward, the pink color observed in the reaction vessel is most likely due to organonitrogen compounds. Day 2 samples included molecules such as pterins and pteridines and the day 4 samples included nitrotoluene molecules. These molecules are most likely the cause of the pink-orange color observed in our reaction vessel. Also, in contrast to Miller’s experiment, our reaction solution stayed a pink-orange color and did not change into a dark red-brown color even after 7 days of running the experiment.

Both the 2 day and 4 day time points included the amino acids serine, phenylalanine, glutamic acid, arginine, leucine, isoleucine and valine. While glutamine, alanine and tyrosine were observed in the 2 day sample, they were not present in the day 4 sample. However, at 4 days post-spark application we did observe histidine, aspartic acid, proline and tryptophan. The emergence of aspartic acid and the absence of alanine in the 4 days sample could be attributed to the pH of the sample mixture. In previous spark discharge experiments it has been noted that acidification of the reaction mixture reduced the yield of amino acids observed at the end of the experiment [8]. In our samples, the pH of the starting solution was near a pH of 12 (average of 11.6), dropped to pH 9 by day 2 and reduced further to pH 8 by day 4. The pH then held at 8 until the end of the experiment. Further analysis of the data showed that after 7 days of running the experiment, the amino acid groupings seen earlier start to break down and the chemical components in the mixture form large multiple ring structures. This in in contrast to the early Earth condition experiments where we still see a fairly complex mixture of amino acids 7 days after the start of the experiment (Table 1 and Table 2). This breakdown of amino acid groupings could be due to the further acidification of the reaction mixture after the 4 days time point.

While our experiments are the first to simulate Titan post-impact conditions, other researchers have completed simulations of Titan atmospheric conditions. Due to the presence of large, complex organic molecules in Titan’s upper atmosphere, Horst *et al.* [17] simulated upper atmospheric conditions on Titan and produced a range of amino acids and nucleotide bases. In particular, they produced cytosine, uracil, thymine, guanine, glycine, alanine and adenine [17]. The only amino acid produced in common between the atmospheric experiments and our experiments was the amino acid alanine. However, these results are exciting in that they provide another source of prebiotic material on Titan.

Overall, our experiments show that it is possible to produce a wide range of amino acids on Titan in post-impact conditions. This finding could have important implications for the chemical evolution and possibly for an origin of life on Titan, either in the past or even persisting to the present day. It is especially interesting that we observed a wider range of amino acids in the Titan condition experiments than in the early Earth condition experiments. These amino acids were also always found in groups of two three and not individually. Even the eventual break down of amino acids observed at the 7 day time point could have implications for life on Titan since the larger carbon ring structures could also be important in the chemical “soup” of life by acting as scaffolds for the building of chains of amino acids. On the other hand they could, however, be just a tar-like substance presenting a dead-end scenario for the development toward further complexity. If the latter scenario is correct, it would mean that the availability of amino acids for further organic synthesis reactions may be severely time-constrained and reactions toward higher prebiological complexity have to set in within a few days after the impact event in order to proceed. The observation that these amino acids are grouped together may provide a hint that such reactions are indeed occurring. Further experiments and analysis of the created organic compounds are needed to resolve this important question.

## 4. Conclusions

The Titan post-impact reaction conditions produced amino acids beginning at least 2 days post application of spark and continued to increase in quantity and complexity (groupings of two to three amino acids were observed) up to 4 days after the spark was applied. The most common amino acids observed were serine, phenylalanine, glutamic acid, arganine, leucine, isoleucine and valine. However, after 7 days of experimental activity the groupings of amino acids seen at day 4 started to break down and form large carbon ring structures. Our results show that not only is it possible to produce amino acids under Titan post-impact conditions, we see different amino acids from those identified in the original Miller conditions. Further experiments using our Miller apparatus will include the addition of sulfur to the reaction mixture as well as trace gases (tholins) found in the atmosphere of Titan.

## References

[1] Miller S.L. (1953). A production of amino acids under possible primitive earth conditions. Science.

[2] Oparin A.I., Lorenz R.D., Lunine J.I. (1968). The Origin of Life.

[3] Lob W. (1913). Uber des Verhalten des Formamids Unter der Wirkung der stillen Entladung, Ein Beitrag zur Stickstoff-Assimilation. Berichte der Deutschen Chemischen Gesellschaft.

[4] Garrison W.M., Morrison J.G., Hamilton A.A., Benson M., Calvin M. (1951). The reduction of carbon dioxide by ionizing radiation. Science.

[5] Kasting J.F., Pollack J.B., Crisp D. (1984). Effects of high CO_2_ levels on surface temperature and atmospheric oxidation state of the early Earth. J. Atmos. Chem..

[6] Kasting J.F. (1993). Early Earth’s atmosphere. Science.

[7] Anderson R., Gathman S., Hughes J., Björnsson S., Jónasson S., Blanchard D.C., Moore C.B., Survilas H.J., Vonnegut B. (1965). Electricity in volcanic clouds. Science.

[8] Cleaves H.J., Chalmers J.H., Lazcano A., Miller S.L., Bada J.L. (2008). A reassessment of prebiotic organic synthesis in neutral planetary atmospheres. Orig. Life Evol. Biosph..

[9] Bada J.L. (2013). New insights into prebiotic chemistry from Stanley Miller’s spark discharge experiments. Chem. Soc. Rev..

[10] Schulze-Makuch D., Grinspoon D.H. (2005). Biologically enhanced energy and carbon cycling on Titan?. Astrobiology.

[11] O’Brien D.P., Lorenz R.D., Lunine J.I. (2005). Numerical calculations of the longevity of impact oases on Titan. Icarus.

[12] Fischer G., Gurnett D.A. (2011). The search for Titan lightning radio emissions. Geophys. Res. Lett..

[13] Horvath G., Skalny J.D., Mason N.J., Klas M., Zahoran M., Vladoiu R., Manole M. (2009). Corona discharge experiments in admixtures of N_2_ and CH_4_: A laboratory simulation of Titan’s atmosphere. Plasma Sources Sci. Technol..

[14] Plankensteiner K., Reiner H., Rode B.M., Mikoviny T., Wisthaler A., Hansel A., Märk T.D., Fischer G., Lammer H., Rucker H.O. (2007). Discharge experiments simulating chemical evolution on the surface of Titan. Icarus.

[15] Scripps Center for Metabolomics. http://metlin.scripps.edu/.

[16] Lavvas P., Yelle R.V., Vuitton V. (2009). The detached haze layer in Titan’s mesophere. Icarus.

[17] Horst S.M., Yelle R.V., Buch A., Carrasco N., Cernogora G., Dutuit O., Quirico E., Sciamma-O’Brien E., Smith M.A., Somogyi A. (2012). Formation of amino acids and nucleotide bases in a Titan atmosphere simulation experiment. Astrobiology.

